# Predicting Protein Folds with Fold-Specific PSSM Libraries

**DOI:** 10.1371/journal.pone.0020557

**Published:** 2011-06-16

**Authors:** Yoojin Hong, Sree Vamsee Chintapalli, Kyung Dae Ko, Gaurav Bhardwaj, Zhenhai Zhang, Damian van Rossum, Randen L. Patterson

**Affiliations:** 1 Department of Computer Science and Engineering, The Pennsylvania State University, University Park, Pennsylvania, United States of America; 2 Center for Computational Proteomics, The Pennsylvania State University, University Park, Pennsylvania, United States of America; 3 Department of Biology, The Pennsylvania State University, University Park, Pennsylvania, United States of America; 4 Department of Biochemistry and Molecular Biology, The Pennsylvania State University, University Park, Pennsylvania, United States of America; 5 Department of Physiology and Membrane Biology, University of California Davis Medical School, Sacramento, California, United States of America; 6 Department of Biochemistry, University of California Davis Medical School, Sacramento, California, United States of America; 7 The Genome Center, University of California Davis Medical School, Sacramento, California, United States of America; Semmelweis University, Hungary

## Abstract

Accurately assigning folds for divergent protein sequences is a major obstacle to structural studies. Herein, we outline an effective method for fold recognition using sets of PSSMs, each of which is constructed for different protein folds. Our analyses demonstrate that FSL (Fold-specific Position Specific Scoring Matrix Libraries) can predict/relate structures given only their amino acid sequences of highly divergent proteins. This ability to detect distant relationships is dependent on low-identity sequence alignments obtained from FSL. Results from our experiments demonstrate that FSL perform well in recognizing folds from the “twilight-zone” SABmark dataset. Further, this method is capable of accurate fold prediction in newly determined structures. We suggest that by building complete PSSM libraries for all unique folds within the Protein Database (PDB), FSL can be used to rapidly and reliably annotate a large subset of protein folds at proteomic level. The related programs and fold-specific PSSMs for our FSL are publicly available at: http://ccp.psu.edu/download/FSLv1.0/.

## Introduction

It has been proposed that the number of distinct native state protein folds is extremely limited [1]. In addition, structure is more conserved than sequence similarity [1]–[3]. Taken together, these attributes underscore the inverse protein folding problem; whereby the vast and varied numbers of primary amino acid sequences that exist in biology occupy a relatively limited number of structural folds. Due to the extreme divergence (≤25% pairwise identity) that can exist between structurally determined (template) sequences and structurally unknown (target) sequences, fold recognition is often compromised. Thus, the crucial information specifying protein structure must be contained in a very small fraction of the amino acid sequence, making the informative points hard to measure. Therefore, any solution to the inverse protein folding problem using template-based modeling must be able to identify these information points and use them to relate targets to appropriate template sequences.

PSSMs (Position Specific Scoring Matrices) are a simple but powerful tool to measure remote homology based on the substitution information in related sequences. It is well-established that PSSMs contain more information than individual sequences [7]–[9]. In previous studies [4]–[6], we demonstrated that well-curated library of PSSMs for a particular protein characteristic (e.g., protein function or structure) and low identity alignment from the library are effective for annotating protein sequences for a specific protein characteristic. In this study, we extend this idea to structural similarity detection. Herein, we report that FSL (Fold-specific PSSM Libraries) is a fast and robust method for fold recognition which works in the “twilight-zone” of sequence similarity. We propose that, with further library development, this method is sufficiently fast that protein sequences can be annotated at proteomic scales.

## Methods

### Fold-specific PSSM Libraries

The power behind our method is derived from user-defined libraries of PSSMs of structurally similar proteins. We take advantage of the increased information content of PSSMs and the speed of BLAST to measure structural similarities among highly divergent proteins. There are three features which make our method distinct from traditional sequence analysis methods. First, we measure target protein sequences with multiple structure-specific PSSM libraries. Second, we quantify low identity alignments, which are traditionally considered statistically insignificant. Third, we consider all relationships (to the same fold and different folds) to extract meaningful signals, which appear to be important for measurements in the “twilight zone” [4]–[6], [10].

**Figure 1:**
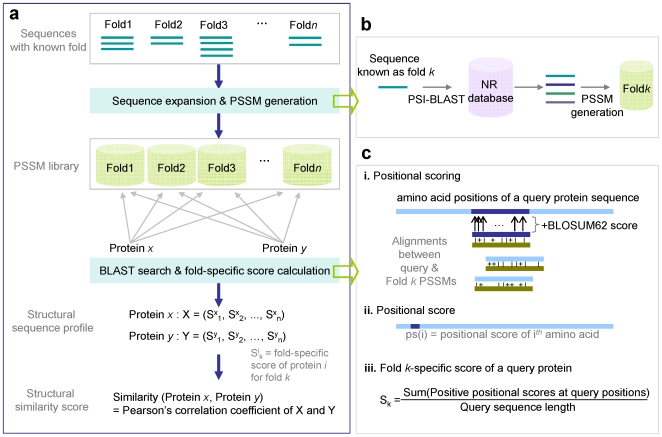
Computational Pipeline. **(a)** Basic pipeline for *FSL* method. For each fold, structurally determined sequences are collected, expanded by PSI-BLAST, and used to generate PSSMs to create a fold-specific library. Each fold-specific library, compiled as rps-BLAST database, can be searched at varying e-value thresholds. Given the alignments returned after filtering by coverage, a fold-specific score for the query is calculated. By repeating this process using different fold-specific libraries, the query protein can be represented as a *structural sequence profile*, which is a vector of fold-specific scores. To calculate *structural similarity score* of two proteins, Pearson's correlation coefficient of their *structural sequence profiles* is calculated. **(b)** Expansion of reference sequences. SCOP domain sequences with known fold are collected, and expanded by PSI-BLAST search against NCBI NR database with each of the reference sequences as a query. After removing redundant or highly similar sequences, PSSMs are generated from the collected sequences by PSI-BLAST for a fold-specific library. **(c)** Calculation of fold-specific scores. Given an alignment between a query and a fold-specific PSSM, each query amino acid is scored with BLOSUM62 score for identical or conserved matches. After scoring with all alignments against PSSMs from the fold-specific library, the fold-specific score of a protein is calculated by dividing the sum of all positive positional scores by a query sequence length.

Our method involves four steps to infer remote structural similarity among proteins (Fig. 1). For these experiments, we generated PSSM library for 1,086 fold (SCOP 1.65) using domain sequences of each SCOP fold as reference sequences. SCOP folds have been hand-curated, making them a reliable resource for building our initial FSL. Importantly, these reference sequences have ≤40% pairwise identity to each other, making them highly divergent. Except in cases where large numbers of reference sequences already exist (e.g. SCOP fold b.1; *Immunoglobulin-like beta-sandwich* fold which already has >1000 sequences), all fold groups were expanded by PSI-BLAST [8] search against NCBI NR database using references sequences of the fold groups as queries. The settings for PSI-BLAST were 3 maximum number of iterations (-j option), 30 maximum number of database sequences returned at each iteration (-b option), 1.0e-6 e-value threshold for including sequences for PSSM generation at each iteration (−h option), and other options remained as default. The sequences similar to the queries (≥90% identity) were removed.

Given the expanded sequences for each fold group, redundant or highly similar sequences (≥40% identity by Needleman-Wunsch algorithm [18]) were also eliminated. Fold-specific libraries for 1,086 fold groups were then constructed by generating PSSMs from the expanded sequences by PSI-BLAST (-j 2 –h 1.0e-6) [19]. Following, fold-specific PSSMs were compiled as a BLAST compatible database [13] (Fig. 1b).

Second, each query sequence is then searched against the fold-specific PSSM libraries using rps-BLAST. The alignments returned from the search are filtered out if they do not satisfy our e-value and coverage thresholds (i.e., alignment length as a function of library PSSM length). In this study, alignments were collected using either of e-value 0.01, no coverage or e-value 10^10^, 80% coverage thresholds. These settings were chosen based on our previous study which demonstrated that both settings provide unique and accurate solutions [20]. Unless otherwise denoted, the results from this study use an e-value 0.01 and no coverage thresholds.

From the alignments to a fold-specific library, a fold-specific score is calculated. For every alignment returned from an rps-BLAST search of a given query against a given fold-specific PSSM library, each amino acid of a query which is identically or positively (non-identical, but conserved) aligned is scored with BLOSUM62 score for the aligned pairs. These scores are summed for each amino acid of the query (i.e., positional score). The fold-specific score for a query protein is calculated as: 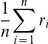
if 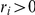 where *n* is the length of a protein sequence and 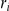 is a positional score of i^th^ amino acid of the protein. Then each query is encoded in a structural sequence profile which is a vector of fold-specific scores (Fig. 1c).

Next, as a quantitative measure of how two proteins are structurally similar (i.e. the structural similarity score), we calculate a Pearson's correlation coefficient between their vectors. Pearson's correlation coefficient between structural sequence profiles *X* and *Y*, *PC(X, Y)*, is calculated as: 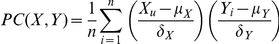
where *n* is the number of measuring folds, and 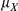
and 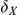
are the average and standard deviation of *X*.

## Results

Initially, we tested the efficacy of our method using the TZ-SABmark, which is a carefully curated benchmark set of fold-specific sequences of remote homology [11]. Each sequence group of TZ-SABmark represents a SCOP fold classification [12] of related sequences with ≤25% pairwise sequence identity. From the original TZ-SABmark, 534 sequences from the first 61 fold groups (avg. length of 135.27
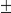
89.39 s.d.) were used as a test set. SCOP domains in TZ-SABmark set were not used as reference sequences for fold-specific library construction. Since we used SCOP domain reference sequences with ≤40% pairwise identity, pairwise identities between TZ-SABmark test sequences and the reference sequences should be also ≤40%.

### Alignment Comparisons and Information Content

**Figure 2:**
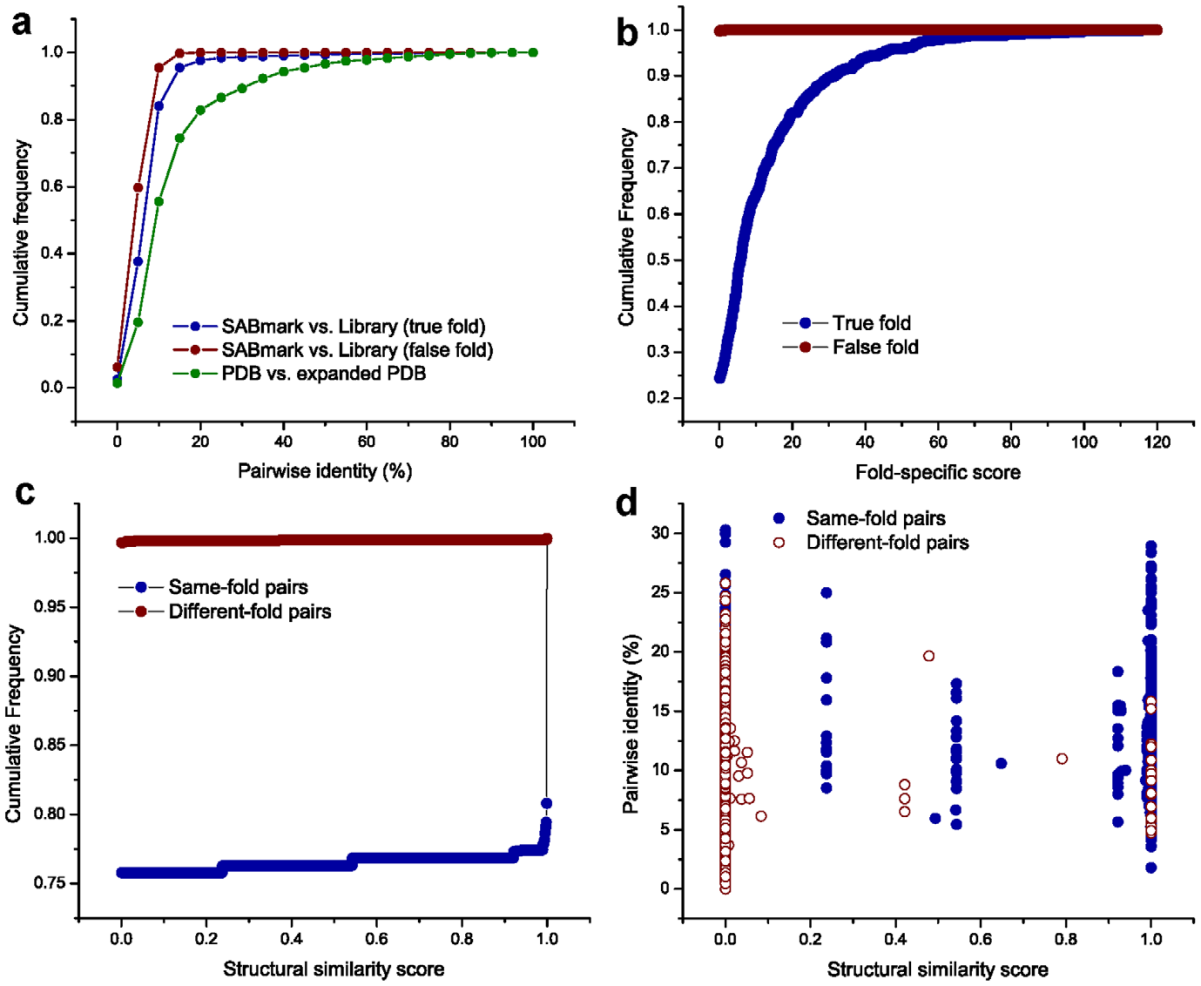
Characterization of Structural Similarity Scores. **(a)** The distribution of percent pairwise identity between pairs of TZ-SABmark sequences and the library sequences of the same-fold (blue) and different-folds (red) and percent pairwise identity between the original PDB sequences and PSI-BLAST expanded sequences (green). All three comparisons demonstrate that nearly all of the sequence alignments reside in the “twilight-zone” of sequence similarity. The pairwise identity was calculated from Needleman-Wunsch global alignments] with BLOSUM62], Gap opening penalty 10, and Gap extension penalty 0.5. **(b)** The distributions of query sequence scores for each fold-specific library. **(c)** Cumulative frequencies of the *structural similarity scores* between pairs of same-fold (blue) and different-fold (red) query sequences. For this measurement, 3,428 same-fold pairs and 65,536 different-fold pairs were measured from 534 sequences. **(d)** *Structural similarity scores* between pairs of same-fold and different-fold query sequences were plotted versus their pairwise sequence identity. This data shows an independent trend between the *structural similarity score* and pairwise identity in the “twilight-zone” of sequence similarity. For different-fold pairs, randomly selected 10,000 data points were plotted. The statistics in the Fig.S1 b, c, and d were obtained given the setting of e-value 0.01, no coverage threshold.

We first evaluated sequence similarity between TZ-SABmark test sequences and the expanded sequences used for building fold-specific libraries. Figure 2a plots cumulative frequency distributions of pairwise identity between pairs of TZ-SABmark test sequences and the sequences from their true- and false-fold libraries. These statistics demonstrate that ∼95% of all same-fold pairs have <20% pairwise identity. Indeed, this distribution is negligibly distinct from comparisons of different-fold pairs. Additionally, we compared the sequence similarity between the SCOP reference sequences and the sequences which were obtained through their PSI-BLAST expansion. The sequences used to define fold-specific libraries are also in the “twilight zone”. Taken together, this indicates that our information source is: (i) derived from low-identity alignments, (ii) improved by including intermediate sequences in the library, and (iii) not due to redundancy.

It is reasonable to consider that a protein would have a larger fold-specific score for its true-fold than for its false-folds; this is confirmed in Figure 2b and demonstrates that our fold libraries are specific. We observe that 99.8% of the query sequences have fold-specific scores 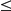
0.1 for different-folds, while only 24.3% of them have scores 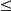
0.1 for same-folds. Given these data, if we annotate each protein by the highest fold-specific score, the folds of 70.8% of TZ-SABmark test sequences can be predicted correctly. Figure 2c shows cumulative frequencies of structural similarity score between pairs of same-fold (blue, 3,428 pairs) and different-fold (red, 65,536 pairs) query sequences. ∼24.2% of same-fold pairs have structural similarity scores >0.1, while only ∼0.2% of different-fold pairs have scores >0.1. Figure 2d plots structural similarity scores between same/different-fold pairs versus their pairwise identity. We observe an independent trend between structural similarity score and pairwise identity whereby true positives distribute to higher structural similarity scores (see Fig. S1 for the statistics of e-value 10^10^, 80% coverage threshold setting).

### Performance Evaluation

To compare our performance for relating structurally related proteins against other benchmarking methods, we utilized receiver operating characteristic (ROC) curve analysis [14]. A ROC curve plots sensitivity versus false-positive rate, where a left-shifted curve is considered more accurate. Sensitivity and false-positive rates are calculated as: Sensitivity  = 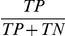, false positive rate  = 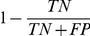, when TP  =  the number of true positives, TN  = the number of true negatives, and FP  =  the number of false positives. SAM-T2K, prof_sim, HHsearch 1.5.0 and FFAS03 are used as benchmark methods [15]–[17].

#### Settings

For SAM-T2K, blastall in NCBI BLAST 2.2.15 is used for the target2k script in the SAM3.5 package for searching the sequence database to collect sequences for HMM generations of the 534 test sequences. When a query sequence is scored given a HMM model by hmmscore, the Smith-Waterman algorithm was used by default. For prof_sim, sequence profiles were generated by PSI-BLAST and profile-profile alignment was done with the local alignment setting. For HHsearch 1.5.0, PSI-BLAST was used for HHsearch to build HMMs of TZ-SABmark test sequences with the setting of –j 5 –h 1.0e-3. The database of TZ-SABmark HMMs were generated and searched for each query HMM with default settings. For all of four benchmark methods, NCBI NR database with 6,419,591 protein sequences was used as a sequence database. FFAS03 was run by a member of Godzik lab [15] to a false-positive rate ∼0.01. In the result of each method, all-against-all comparison of TZ-SABmark test sequences were performed, and for each sequence, all other sequences were sorted by structural similarity score (in case of our method) or e-value/p-value (in case of benchmarking methods) for ROC curve analysis. In all cases, the settings used were chosen as to give each method the best chance of performing well.

#### Results

**Figure 3:**
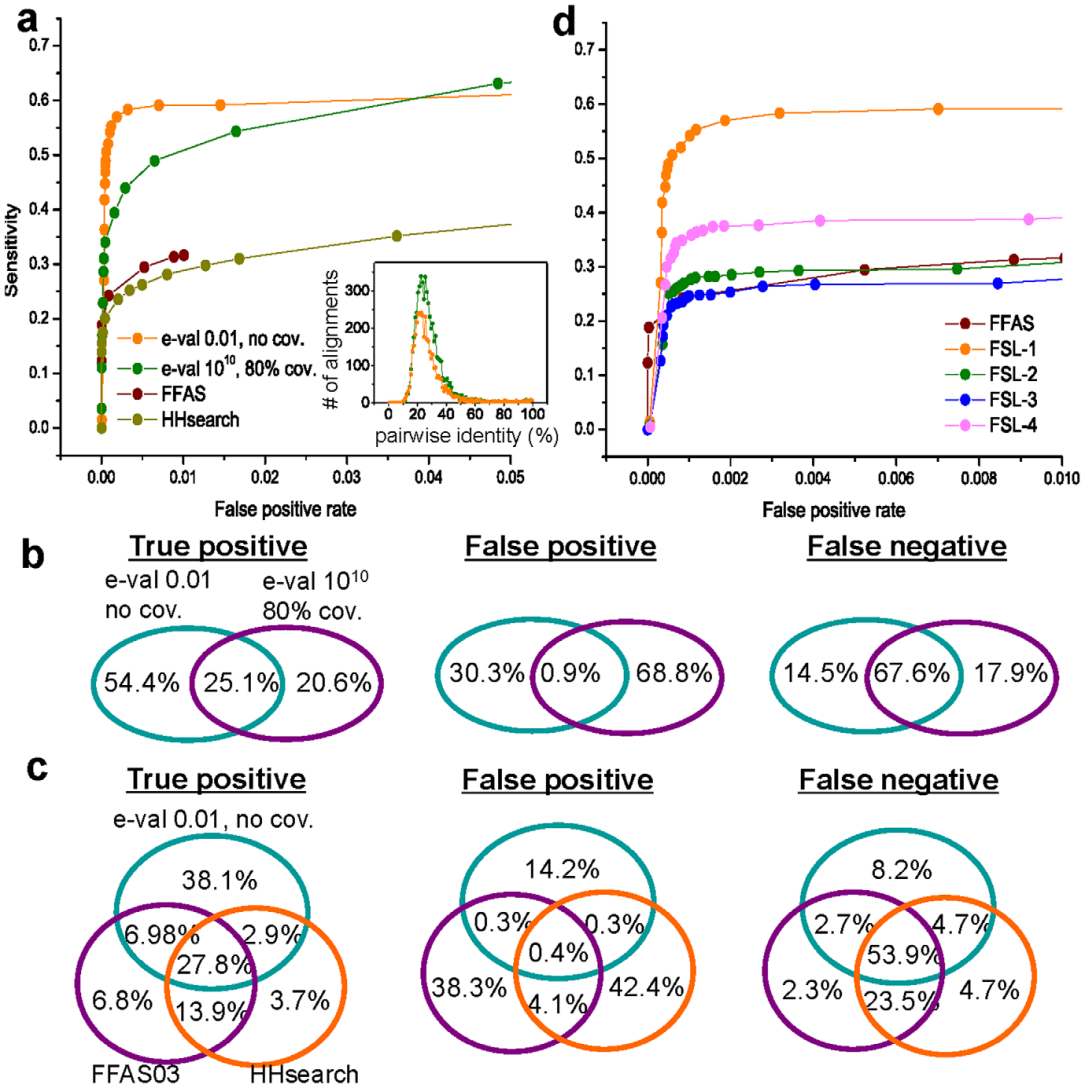
Fold Recognition Performance and Comparative Statistics. **(a)** Comparison of ROC curves of *FSL* with two different settings, FFAS03 and HHsearch. Pairwise identities of the alignments between queries and the PSSMs from their true fold-specific library, which were collected with different e-value and coverage thresholds, are shown (*inset*). **(b)** Comparison of true-positive, false-positive, and false-negative pairs in top-9 result (sequences returned with the highest 9 structural similarity scores or the lowest 9 e-value/p-value for each of TZ-SABmark queries) of *FSL* at two different settings of e-value 0.01 and 10^10^. The numbers of true-positive, false-positive, and false-negative pairs predicted by *FSL* of e-value 10^10^ are 2,616, 2,190, and 4,240, respectively. **(c)** Comparison of true-positive, false-positive and false-negative pairs in top-9 result of *FSL* (e-value 0.01, no coverage), FFAS03, and HHsearch. The numbers of true-positive pairs predicted by *FSL*, FFAS03, and HHsearch are 2,773, 2,030, and 1,769, respectively. The numbers of false positive pairs are 980, 2,776, 3,037, while the numbers of true negative pairs are 4,069, 4826, and 5,087 (*FSL*, FFAS03, and HHsearch respectively). **(d)** Comparison of ROC curves of *FSL* given fold-specific libraries built with different sets of reference sequences (e-value 0.01, no coverage) and FFAS03. FSL-1 is the result using PSSM libraries built from the SCOP reference sequences only after removing TZ-SABmark test sequences. FSL-2 and FSL-3 are the results using PSSM libraries built from SCOP domains whose sequence identity is <30% and <25% to TZ-SABmark sequences. FSL-4 is the result using PSSM libraries built from SCOP domains whose sequence identity is <25% to TZ-SABmark sequences at less stringent settings for PSI-BLAST for expansion.

In Figure 3a, we compare ROC curves of our method with two different settings (e-value 0.01, no coverage and e-value 10^10^, 80% coverage thresholds, see Fig. S2b for the results of different threshold settings) versus two traditional fold recognition methods (FFAS03 and HHsearch [15], see Fig. S2a for prof_sim and SAM-T2K [16], [17]). The results demonstrate that our method in both settings outperform these benchmarking methods. The sensitivity of our method using only statistically significant alignments from rps-BLAST (e-value 0.01, no coverage) is ∼0.6 at false positive of 0.01. At the setting of e-value 10^10^ and 80% coverage, we obtain similar sensitivity at a false positive rate ∼0.04, but its sensitivity increases up to ∼0.7 at a false positive rate 0.1 due to the additional alignments obtained. Intriguingly, the alignments obtained from both filtering strategies reside in the “twilight zone” (Fig. 3a inset).

Figure 3b quantifies the independence between predictions of FSL with two different settings (e-value 0.01, no coverage vs. e-value 10^10^, 80% coverage) for true-positives, false-positives, and false-negatives. Interestingly, we observe a significant number of unique true-positive pairs at both e-value settings. This suggests that comparative measurements are likely to be useful for the identification of true-positive pairs. We made the same comparison between our method (e-value 0.01, no coverage), FFAS03, and HHsearch (Fig. 3c, see Fig. S3 for comparisons using e-value 10^10^, 80% coverage threshold). The diagrams indicate that FSL obtain more unique true-positive pairs and false-negative pairs while predicting fewer false-positive pairs. The most dramatic increase occurs between unique true positives whereby FSL obtain 5.6 fold increase over FFAS03 and a 10.3 fold increase over HHsearch.

Additionally, we tested how our method performs when building fold-specific libraries only using reference sequences which have <30% (FSL-2) and <25% (FSL-3, FSL-4) sequence identity to TZ-SABmark test sequences (Fig. 3d). As expected, as we limit references sequences by their similarity to TZ-SABmark test sequences, our performance degrades (FSL-1, FSL-2, FSL-3). Instead of only using SCOP domains whose sequence identity is <25% to the test sequences as references, we allowed PSI-BLAST to return a larger number of sequences while expanding reference sequences using more relaxed settings (-j 5, −b 60, −h 1.0e-3). By this simple change of PSI-BLAST setting for expansion, we could build sensitive FSL, which outperforms all benchmarking methods, with this very limited set of reference sequences (FSL-4).

### Applications for Fold Classification

**Figure 4:**
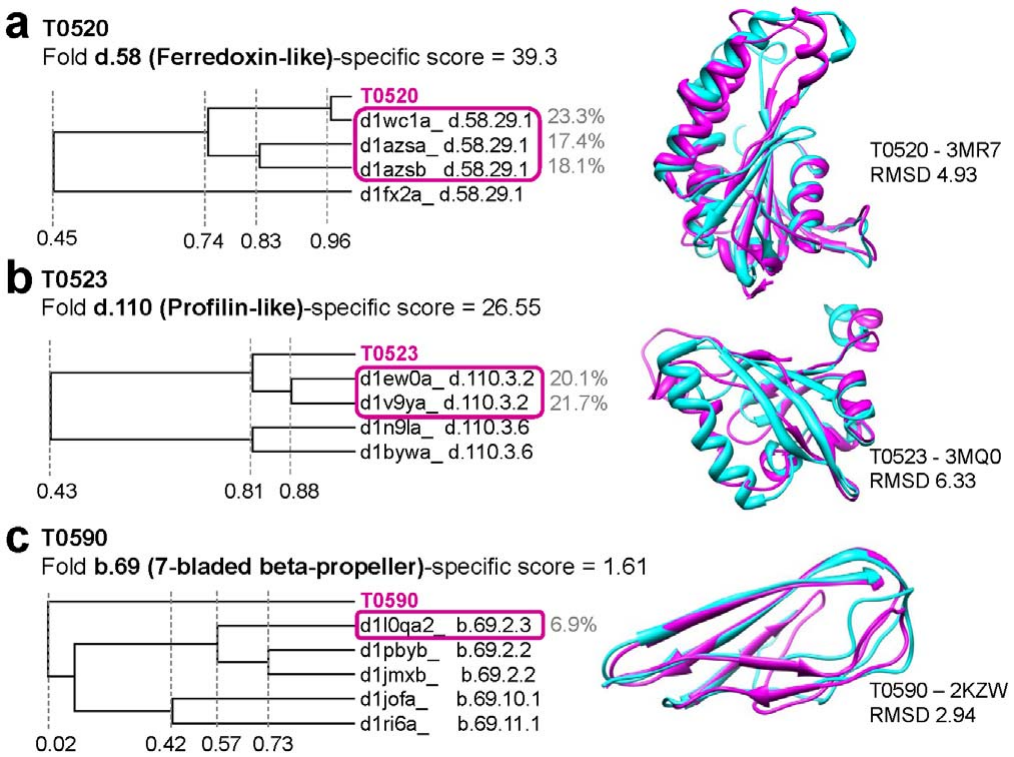
Structure prediction of CASP9 targets. The structures of the three targets T0520, T0523, and T0590 from CASP9 were predicted based on the folds predicted *FSL*. First, the fold of each target was determined as the fold with the highest fold-specific score. To select the best template structures from the fold, all SCOP domains in the fold (SCOP 1.75) and the target were represented in a vector of (percent identity 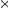 percent coverage) score of an alignment to each PSSM in the fold-specific library and then performed hierarchical clustering using Pearson's correlation coefficient as a similarity metric. The SCOP domains which have high Pearson's correlation coefficients to the target were selected as template structures for template-based structure modeling (purple boxes in a dendrogram). Sequence identity between the SCOP domains and the target is in the twilight zone (grey text). The sequences of the selected SCOP domains and the target were aligned using MUSCLE. Given the alignment, Modeller was used to predict a structure. Structural superposition of each target to its experimental structure is given (cyan: experimental structure, purple: our model).

Based on the promising results described above, we sought to perform a forward-engineering and blind experiment using targets from the 9th Critical Assessment of Techniques for Protein Structure Prediction (CASP) competition that recently finished. We present here a few target results (T0520, T0523, and T0590 in Fig 4 a, b, & c respectively) only to demonstrate that our approach can be applied to template-based structure modeling. These challenging proteins were provided as “human/server targets” (i.e., Template Based Modelling {TBM}) because of their weak similarity to known structures. All of our results for the competition are provided in Table S1 a–d.

We first determined which of the SCOP fold libraries had the highest fold-specific score with the target. To select the best template, we create a structural sequence profile for the representatives of the best fold and then perform hierarchical clustering using Pearson's correlation coefficient as a similarity metric. The SCOP domains with high Pearson's correlation coefficients to the target are then used as templates for template-based structure modeling (purple boxes). Sequence identity between the SCOP domains and these targets are highly divergent (grey text). Following clustering, the sequences of the selected SCOP domains and the target were aligned using MUSCLE [21] and threaded models were generated using Modeller [22]. We observe that our backbone models for these three targets accord well to the crystal structures as shown in the Fig 4. These results suggest that this application holds promise for structural modeling.

We were unable to model all CASP9 targets as successfully as the targets described above. When compared to FFAS03n and HHPredA, we obtained a “best template” as determined by the CASP9 curators for 24/43 human/server targets, while FFAS03n obtained 26/43 best templates and HHPredA obtained 33/43 best templates. When we examined our data for the 19 unsuccessful targets, 18/19 targets had less than 6 PSSMs in their FSL (Table S1a–d). Thus, our performance can be improved by making more comprehensive FSLs that include all PDB structures.

## Discussion

In this manuscript we reveal the power of FSL for fold recognition in the “twilight-zone” of sequence similarity. Our results support the hypothesis that FSL provides a robust user-defined structural modeling application. This is supported by several key findings from our measurements: (i) “twilight-zone” pairwise alignments are informative (Fig. 2), (ii) they outperform multiple benchmarking methods in TZ-SABmark by providing more unique true-positive pairs (Fig. 3), and (iii) they are capable of reconstituting structural fold classifications, including sub-fold groupings (i.e., SCOP superfamilies) that are not encoded in the PSSM library (Fig. 4). A number of broad implications can be derived from this study.

We previously reported that low-identity alignments are a rich source of information, which can be used to unmask the fundamental properties of proteins, including protein structure, function, and evolution using simple arithmetic [4], [5], [10]. We take advantage of the information content provided by PSSMs to increase the signal-to-noise ratio inherent to low-identity alignments. In addition, we demonstrate that a coverage threshold is an effective filter of noisy alignments (Fig S2c). When fold-specific scores are encoded into a vector (i.e., structural sequence profiles), multiple data mining algorithms can be used reliably to measure fold attributes.

We also evaluated the performance of FSL to relate divergent structural folds of test sequences by correlating their structural sequence profiles, which are generated for test sequences after measured with fold-specific PSSM libraries. When compared to popular profile-based algorithms such as FFAS03, HHsearch, SAM-T2K, and prof_sim, FSL obtains a significant portion of unique true-positive pairs and reduced false-positives. Taken together, this underlies our increased performance. Interestingly, all methods including FSL recover a substantial number of unique pairs. While relating these unique pairs (outside of our own) is difficult, if a scoring function could be assigned to unique information obtained from each server (e.g. similar to the MULTICOM or RAPTOR algorithms [22], [23], it is likely that further improvements could be achieved.

Considering the current genomic explosion of sequences, fold recognition methods are needed as they are a true watershed in Biology. Based on the results presented here, conversion and PSI-BLAST expansion of the PDB into fold-, superfamily-, and family-specific PSSM libraries would, in theory, synergize and improve structural modeling in general. In this study, we used FSLs comprised of sequences exclusively derived from SCOP fold classifications. Thus, the current weakness of our method is our incomplete PSSM fold-specific libraries. Future work is aimed at expansion and improvement of these libraries using all available information in structural databases.

## Supporting Information

Figure S1 **Characterization of Structural Similarity Scores given e-value 10^10^ and 80% coverage threshold. (a)** The distributions of query sequence scores for each fold-specific library. 97.3% of the query sequences have fold-specific scores < = 0.1 for different-folds, while only 20.0% of them have scores < = 0.1 for same-folds. **(b)** Cumulative frequencies of the *structural similarity scores* between pairs of same-fold (blue) and different-fold (red) query sequences. 66.3% of same-fold pairs have *structural similarity scores* >0.1, while 39.7% of different-fold pairs have scores >0.1. For this measurement, 3,428 same-fold pairs and 65,536 different-fold pairs were measured from 534 sequences. **(c)** *Structural similarity scores* between pairs of same-fold and different-fold query sequences were plotted versus their pairwise sequence identities. This data shows an independent trend between the *structural similarity score* and pairwise identity in the “twilight-zone” of sequence similarity. The data points of randomly selected 10,000 different-fold pairs were plotted.
(TIFF)

Figure S2 **Fold Recognition Performance of** ***FSL*** **with Different Settings Given 1,086 fold-specific libraries. (a)** Comparison of ROC curves of *FSL* with two different settings (of e-value 0.01, no coverage and e-value 10^10^, 80% coverage), FFAS03, HHsearch, prof_sim, and SAM-T2K **(b)** Comparison of ROC curves of *FSL* at different coverage thresholds when e-value threshold is fixed at 10^10^.
(TIFF)

Figure S3 **Comparison of true-positive, false-positive and false-negative pairs in top-9 (** ***FSL*** **of e-value 10^10^, 80% coverage threshold, FFAS03, and HHsearch1.5.0).** The numbers of true-positive pairs predicted by *FSL*, FFAS03, and HHsearch1.5.0 are 2,616, 2,030, and 1,769, respectively. The numbers of false positive pairs are 2,190, 2,776, 3,037, while the numbers of true negative pairs are 4,240, 4,826, and 5,087 (*FSL*, FFAS03, and HHsearch1.5.0 respectively).
(TIFF)

Table S1 **60 CASP9 targets for the Human/Server prediction.** CASP9 released 60 targets for the Human/Server prediction. Tertiary structure predictions are divided into two categories namely; “Template based modeling” category which include domains where a suitable template is identified that covers all or nearly the entire target, and “Template free modeling” category which include models of protein for which no suitable template or only a small portion of target is identified. The best template for the target is picked based on the GDT_TS (Global Distance Test_Total Score) between the aligned CA atoms in template and the experimental structure in sequence-independent superposition under 5Å distance cutoff. All the targets are separated into columns and have listed the best templates for all the targets used by CASP. A comparison table is constructed with three main assessors namely WACLabs, FFAS03n & HHpredA in the selection of the best template. **(a)** the list of 24 successful targets predicted by WAC Labs. All the three assessors WACLabs, FFAS03n and HHpredA picked the best template (Fold recognition) used in CASP2010 (colored blue). Here, HHpredA have identified multiple structures to model their target, whereas WAC Labs and FFAS03n have used a maximum of only 3 templates. **(b)** the list of 19 unsuccessful targets predicted by WAC Labs. We (WAC Labs) were unable to predict the best template when compared to HHPredA and FFAS03n who were successful in identifying 11 and 8 template structures out of 19 targets (colored blue) respectively. We selected a poor template with low fold specific score due to our incomplete fold specific library. **(c)** the list of 19 unsuccessful targets predicted by WAC Labs with respect to the PSSMs generated. For the 19 targets we were unable to classify, we have minimal information for 12 targets and 7 targets were not encoded in our FSLs. For the 12 targets that were present in our library (colored red), our fold specific library does not contain sufficient PSSMs to generate useful scores. (d) the list of 17 miscellaneous targets. Out of 17 targets, 6 were cancelled and 11 are modeled by “Template free modeling” (TFM). T0550 and T0608 (colored blue) are exceptions where part of the template region is used to model a part of the protein and later are modeled by template free modeling.
(DOCX)
