# Supplementary figures and images for: Predicting Protein Folds with Fold-Specific PSSM Libraries
Source: PLoS One. 2011 Jun 16;6(6):e20557. doi: 10.1371/journal.pone.0020557 (PMC3116844; doi:10.1371/journal.pone.0020557)

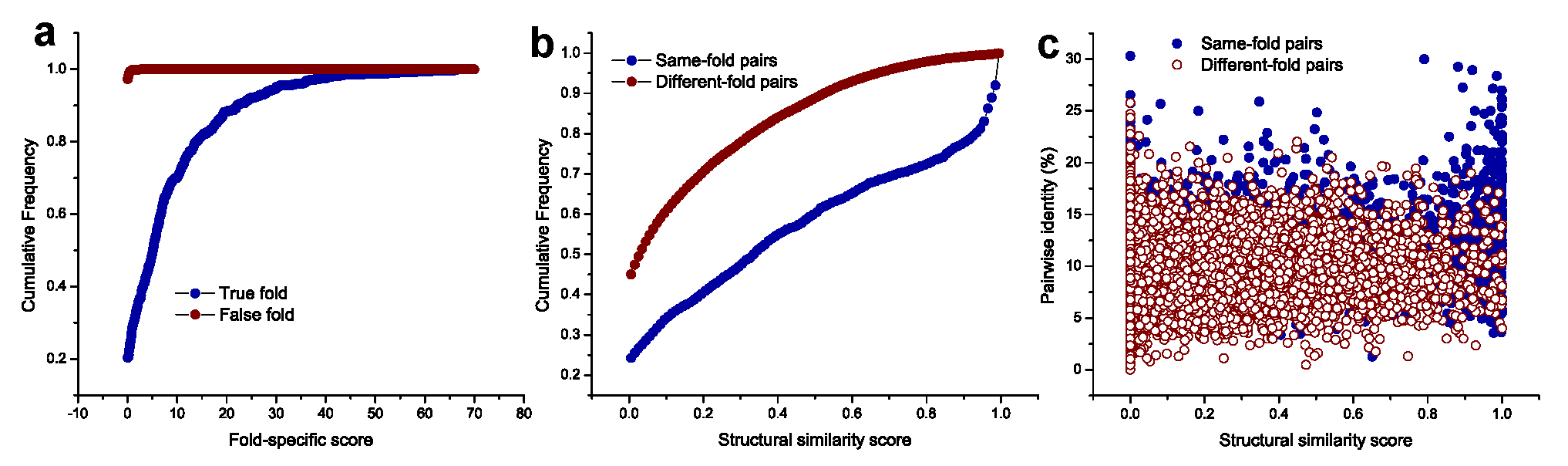

Supplement: Figure S1 — Characterization of Structural Similarity Scores given e-value 1010 and 80% coverage threshold. (a) The distributions of query sequence scores for each fold-specific library. 97.3% of the query sequences have fold-specific scores < = 0.1 for different-folds, while only 20.0% of them have scores < = 0.1 for same-folds. (b) Cumulative frequencies of the structural similarity scores between pairs of same-fold (blue) and different-fold (red) query sequences. 66.3% of same-fold pairs have structural similarity scores >0.1, while 39.7% of different-fold pairs have scores >0.1. For this measurement, 3,428 same-fold pairs and 65,536 different-fold pairs were measured from 534 sequences. (c) Structural similarity scores between pairs of same-fold and different-fold query sequences were plotted versus their pairwise sequence identities. This data shows an independent trend between the structural similarity score and pairwise identity in the “twilight-zone” of sequence similarity. The data points of randomly selected 10,000 different-fold pairs were plotted. (TIFF) [file pone.0020557.s001.tiff]

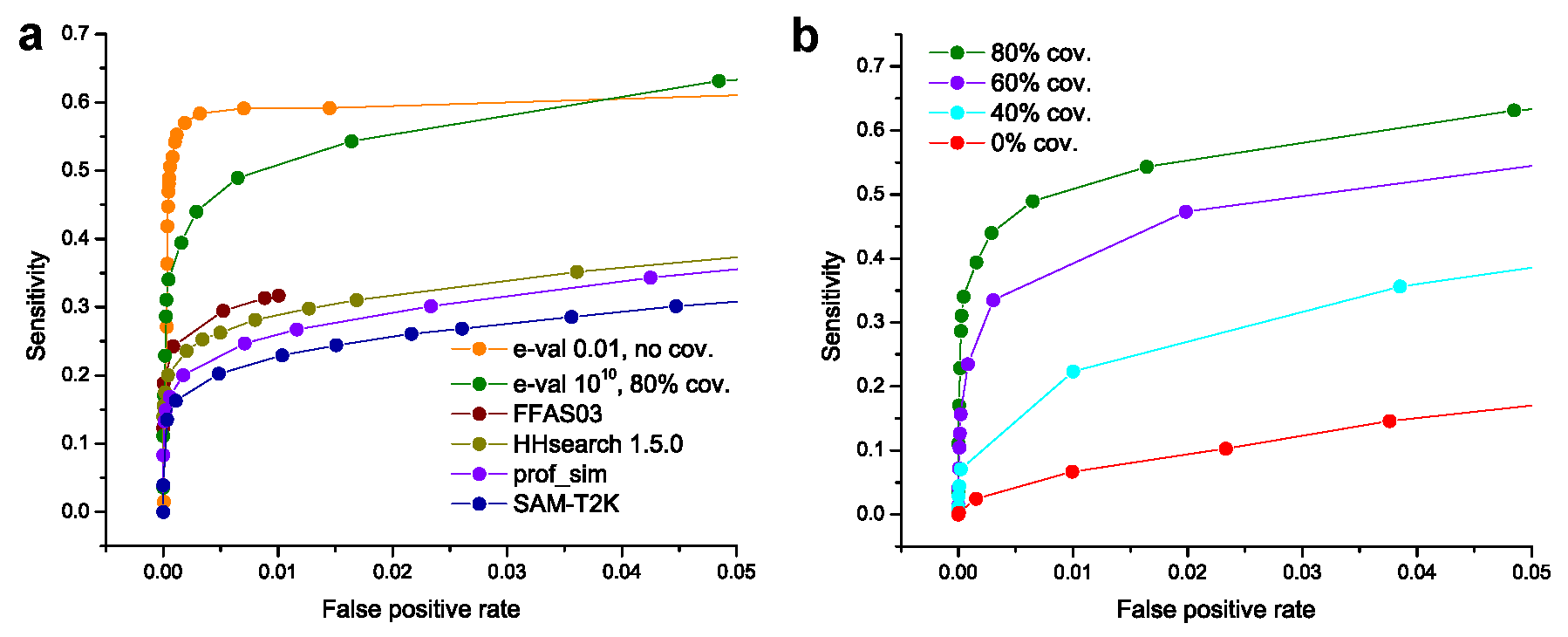

Supplement: Figure S2 — Fold Recognition Performance of FSL with Different Settings Given 1,086 fold-specific libraries. (a) Comparison of ROC curves of FSL with two different settings (of e-value 0.01, no coverage and e-value 1010, 80% coverage), FFAS03, HHsearch, prof_sim, and SAM-T2K (b) Comparison of ROC curves of FSL at different coverage thresholds when e-value threshold is fixed at 1010. (TIFF) [file pone.0020557.s002.tiff]

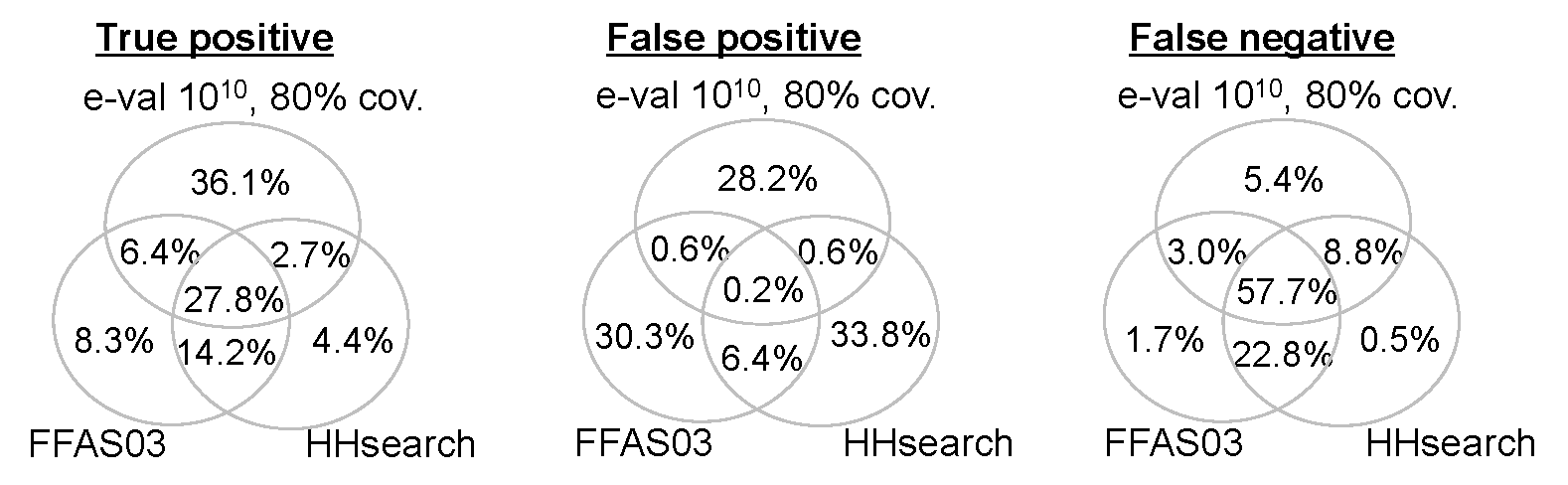

Supplement: Figure S3 — Comparison of true-positive, false-positive and false-negative pairs in top-9 ( FSL of e-value 1010, 80% coverage threshold, FFAS03, and HHsearch1.5.0). The numbers of true-positive pairs predicted by FSL, FFAS03, and HHsearch1.5.0 are 2,616, 2,030, and 1,769, respectively. The numbers of false positive pairs are 2,190, 2,776, 3,037, while the numbers of true negative pairs are 4,240, 4,826, and 5,087 (FSL, FFAS03, and HHsearch1.5.0 respectively). (TIFF) [file pone.0020557.s003.tiff]
