# Supplementary material for: Predicting Protein Folds with Fold-Specific PSSM Libraries
Source: PLoS One. 2011 Jun 16;6(6):e20557. doi: 10.1371/journal.pone.0020557 (PMC3116844; doi:10.1371/journal.pone.0020557)
Supplement: Table S1 — 60 CASP9 targets for the Human/Server prediction. CASP9 released 60 targets for the Human/Server prediction. Tertiary structure predictions are divided into two categories namely; “Template based modeling” category which include domains where a suitable template is identified that covers all or nearly the entire target, and “Template free modeling” category which include models of protein for which no suitable template or only a small portion of target is identified. The best template for the target is picked based on the GDT_TS (Global Distance Test_Total Score) between the aligned CA atoms in template and the experimental structure in sequence-independent superposition under 5Å distance cutoff. All the targets are separated into columns and have listed the best templates for all the targets used by CASP. A comparison table is constructed with three main assessors namely WACLabs, FFAS03n & HHpredA in the selection of the best template. (a) the list of 24 successful targets predicted by WAC Labs. All the three assessors WACLabs, FFAS03n and HHpredA picked the best template (Fold recognition) used in CASP2010 (colored blue). Here, HHpredA have identified multiple structures to model their target, whereas WAC Labs and FFAS03n have used a maximum of only 3 templates. (b) the list of 19 unsuccessful targets predicted by WAC Labs. We (WAC Labs) were unable to predict the best template when compared to HHPredA and FFAS03n who were successful in identifying 11 and 8 template structures out of 19 targets (colored blue) respectively. We selected a poor template with low fold specific score due to our incomplete fold specific library. (c) the list of 19 unsuccessful targets predicted by WAC Labs with respect to the PSSMs generated. For the 19 targets we were unable to classify, we have minimal information for 12 targets and 7 targets were not encoded in our FSLs. For the 12 targets that were present in our library (colored red), our fold specific library does not contain suff [file pone.0020557.s004.docx]

**Table S1**

**(a)**

|  | TARGET | PDB native | Residues | Best Templates used in CASP2010 | WAC Labs | FFAS03n | HHpredA |
| --- | --- | --- | --- | --- | --- | --- | --- |
| 1 | T0515 | 3MT1 | 365 | **2yyxA**, 3c5qA, 2qghA, **1twiA**, 2p3eB, **1tufA**, 2j66A, **2o0tC**, 1ko0A, **1knwA** | **1knwA, 1twiA** | **1tufA** | 2qghA, **2yxxA 2o0TA,** 2p3eA 2j66A, **1twiA** 2pljA, 1f3tA |
| 2 | T0517 | 3NO6 | 229 | **2qs7A**, **1jx7F**, 1l1sA, 2hybH**, 2pd2A**, 2hy5C, 2d1pH | **2qs7A** | **2qs7A** | **2qs7A, 2d1pB 1jx7A ,** 2fb6A 2hy5B, 1l1sA **2d1pA , 2pd2A** |
| 3 | T0520 | 3MR7 | 189 | 1dz5B, 1nu4A, 1m5pC, 3k0jD, 3l3cC, 3hhnD, 1m5vC, 2nz4A, 1urnB, **1azsA, 1ybtA** | **1azsA,** 1wc1A, | **1ybtA** | 2w01A, **1ybtA** 1y10A, 1wc3A 2wz1A, **1azsA** 3et6A, 1fx2A |
| 4 | T0523 | 3MGO | 120 | 3lyxB, 2gj3B, 3f1nA, 3h7wA, 3h82A, 3ewkA, 2vlgC, 3gdiA, 2a24A, **1ewoA** | **1ewoA,** 1v9yA | **2z6cA** | 3lyxA, 3ewkA 2gj3A, 2wkqA **2z6dA,** 3f1pB 3ewkA, 2v0uA |
| 5 | T0526 | 3NRE | 290 | 1dz5B, 1audA, 1m5oF, **1sooA, 1lurA,** 1vcn5A, 1m5kF, 2oj3A, 1vbyA, 1m5pC | **1sooA, 1lurA** | 3dcdA | 1z45A, 3k25A 1snzA, **1lurA** 1ygaA |
| 6 | T0543 | 2XRG | 887 | 2rh6A, **2gsnA**, 2gsuA, **2gsoA**, 1ei6B, 2qzuA, 3ismB, 1e2sP, 1e33P, 1e3cP | **2gsnA,** 1vr7A | **2gsnA,** 1zm8A, 2vj2A | **2gsoA, 3ismA** 1zm8A, 1g8tA |
| 7 | T0547 | 3NZP | 611 | **1twiD**, 1tufB, 3c5qA, **2qghA**, 1hkvA, 2o0tB, **2yxxA**, 1hkwB, 2p3eA, 2j66A | **1twiD, 2yxxA, 2qghA** | **2p3eA** | **2o0tA** ,7odcA **1twiA** |
| 8 | T0566 | 3N72 | 156 | **1usvB**, **1usuB**, 1bp1A, 1ewfA, 2obdA, 3h4zB, 2rgqA, 2rckA, 3l6iA, 2vdfA | **1usvB** | **1usuB** | **1usuB** |
| 9 | T0580 | 3NBM | 105 | 2zwmB, 2qsjB, 1morA, 3idoA, 1nxxA, 3f6pA, **1iibA**, 3mf4A, 3h5lB, **1e2bA** | 1vkrA, **1iibA** | **1e2bA** | **1e2bA** ,1tvmA |
| 10 | T0581 | 3NPD | 136 | 2qx2A,3ib5A,1avqA,2ch6C,3ieuA,1p4dB,1wpmB,2q7uB, **1wf3A , 1w1wA,** 2w45B | 2ezlA, **1w1wA** | 1l9uD | 3e0jB, 1q0vA **1iwlA** |
| 11 | T0582 | 3O14 | 222 | **1sfnA, 2qdrB**, 2h0vB, 1y3tB, 1sq4B, 1juhA, 1rc6B, 2phdD, **1sefA**, 1sq4A | **1sefA** | **2qdrA** | **1sfnA,** 3balA 2o1qA, 1yhfA 3cjxA |
| 12 | T0584 | 3NF2 | 352 | **1wy0A, 1wmwB,** 3krpD, 3kroA, 3kraD, 3krfA, 3lvsB, **3mzvB**, 3m0gB, **2q80A** | 1v4eA, **2q80A** | **1wy0A** | **1wy0A,** 3lmdA 3n3dA, **3mzvA** 1rqjA, 3lsnA **1wmwA** |
| 13 | T0588 | 3NFV | 400 | 3ew4A, 3evlA, 1hv6A**, 1qazA**, **3evhB, 3a0oA**, 3aflA, 2wdaA, 1rwhA, 1rwgA | **1qazA** | **3evhA** | **1qazA ,3a0oA** |
| 14 | T0590 | 2KZW | 137 | 2hs0G, 3fepG, 3hrzB, 2winH, 2qkiB, 2a73B, 2wiiB, **1loqA**, 2iceB, 2qkiE, 2hr0B | **1loqA** | 1wgoA | **1l0qA,** 1dbgA 2c4xA |
| 15 | T0592 | 3NHV | 144 | **3flhB, 3ilmA**, 1bohA,1dp2A, 1boiA, 1rhsA, 2oraA, 1orbA, 1rhdA, **2hhgA** | **2hhgA** | **3ilmA** | **3flhA,** 1yt8A **3ilmA,** 1wv9A 3gk5A |
| 16 | T0594 | 3NI8 | 140 | 1z94C, 2il5A, **1xuvA**, 2nn5A, 1xfsB, **3eliA**, 3ie5A, 2ns9A, 3c0vB, 2flhD, 3kb3A | **1xuvA, 3eliA** | 1x53A | 1x53A, 2il5A **3eliA** |
| 17 | T0596 | 3NI7 | 213 | 3c07A, 3lwjA, 3lhqB, 2np5C, **2hjyA, 2oi8A**, 2g3bB, 3crjB, 1rktB, 2uxpA, 1u9oA | **2hjyA, 2oi8A** | **3c07A** | **3c07_A** ,2w53_A |
| 18 | T0610 | 3OT2 | 186 | **1wdjA**,3ijmB,3cmmA,2abrB,1y8qC,1dc1A,1rxxD,1o17D,2aciC,3kydA | **1wdjA** | **1wdjA** | **1wdjA** |
| 19 | T0619 | 3NRW | 111 | **1z19B**, 1z1gC, 1z1bA, 3lysD, 1p7dB, **1a0pA**, **2oxoA**, 2a3vB, 2aqlA, 3crxA | **1aopA** | **1a0pA** | **1z19A, 1a0pA** 2khqA, 2keyA **2oxoA,** 2kobA 2kkpA |
| 20 | T0622 | 3NKL | 138 | 1ys4A, 3ikvA, 3il2B, 3iktA, **2dt5A**, 2nu9D, 1jkjA, 3hskA, 1eudA, 1nvmD | **2dt5A** | 1xcbA | 2py6A, **2dt5A** |
| 21 | T0627 | 3OQL | 261 | **1rcwA**, 1rtwB, **3hlxA**, 1otwA, 3bjdA, 2gm8C, 1otvB, 2f2gB, 3hmlB, 2q4xB | **1rcwA** | **1rcw_A** | 2qcxA, **3hlxA** 1z72A, **1rcwA** 3mvuA, 3bjdA 3ibxA |
| 22 | T0629 | 2XGF | 216 | **1ocyA**, 1odiB, 2ztbA, 2x44D, 2j60A, 1ea0B, 2nn1A, 2j5zC, 2it4A, 3iszA | **1ocyA** | **1ocyA** | **1ocy_A** 1pdi_A 2fl8_A |
| 23 | T0630 | 2KYT | 132 | **2if6B**, 3kw0B, 3ebqA , **3h41A**, **2evrA**, 2fg0A, 2hbwA, 2cagA, 2x4wA, 3mo8A | **2evrA** | **2if6A** | **2if6A , 3h41A** 3gt2A |
| 24 | T0643 | 3NZL | 83 | 2a6cA,2h8rB, **1ic8B**, 3djlA, 1vz0B, 2ppxA, 2o4aA, 1r71C, 1umqA, 3g5gN, **2ofyA** | **2ofyA**, 1y7yA | 1fuiA | 1lmb3 3kz3_A 2wiuB |

**(b)**

|  | TARGET | PDB native | Residues | Best Templates used in CASP2010 | WAC Labs | FFAS03n | HHpredA |
| --- | --- | --- | --- | --- | --- | --- | --- |
| 1 | T0529 | 3MWT | 529 | 2guiA, 2idoA, 1j54A, 1j53A, 2d5rA , **1wljA, 2p1jA,** 3hfxA, 3kk2A, 3ithA | 2g64A, 2ghvE, 1qu9A | 3h0gA | 3g8rA, **2p1jA 1wljA,** 1a92A 2gbzA |
| 2 | T0544 | 2L3W | 135 | 2ffhA, 3cucA, 2hpsA, 1sl8A, 3iytA, 1z6tA, 1df0B, 3glfF, 2x38A, 3iukA | 1ow1A, 2jo7A | 1l9uD | 1yc9A, 1w3wA 3k44A, 1oyiA 2zfdA |
| 3 | T0558 | 3NO2 | 294 | 3fvzA, 3li3A, 2gvuA, 3hlhB, 2gvwA, 3fw0A, 2iatA, 3hliC, 3elqA, 3g4hA, **1q7fA** | 1w6sA, 1flgA 1kb0B | **1q7fA** | 2z2nA, 3dsmA **1q7fA** |
| 4 | T0562 | 2KZX | 123 | 2qq4F, 1su0B, 3lvlA, **3lwxA,** 3hrdG, 1n63C, 1n5wC, 1n60C, 1n61C, 1ffvF | 1ycsB | **3lwxA** | **3lwxA,** 1qo8A |
| 5 | T0564 | 2LOC | 89 | 3hs0C, 1h9mA, 1h9jA, 3gd7A, 1h9rA, 1h9sB, 2r6gB, 1o7lB, 1h9kA, 3fh6A | 1g9lA,1nmrA | 2vsqA | 1wjjA, 2jysA |
| 6 | T0568 | 3N6Y | 158 | 2jhtA, 1qvyB, 1fsoA, 1ft3B, 1kmtA, 1cc0E, 2jhuA, 2jhsA, 2jhyA, 1fstB | 1d4tA, 1i3zB | 2pn5A | 2pn5A, 2p9rA |
| 7 | T0569 | 2KYW | 79 | **3i57B**, 3lyyA, 2pz4A, 3eg9B, 1r6vA, 3hn5B, 1hzlA, 1kbvC, 1g1oB, 5ttrF | 2bzvA, 2q60A, 1rqpA | **3i57A** | **3i57A,** 2kvzA |
| 8 | T0574 | 3NRF | 126 | 2vztA, 1lmiA, 2vzuB, 2vzoA, 2xo9A, **3cfuA**, 1yewI, 2x09B | 1i8dA | 2f1eA | 1xq4A, **3cfuA** |
| 9 | T0576 | 3NA2 | 172 | 2j0pA, 3fm2B, 2j0rA, 2oviD, 2ph0A, 1u9tA, 3gyoA, 3fssA, 3gypA, 3dbaA | 1mtyG | 2kkuA | 3ig3A, 3hm6X 3h6nA, 2r2oA 3kuzA |
| 10 | T0578 | 3NAT | 164 | 1xy3F, 2ic0A, 3lwtX, 1r56A, 3f2mA, 1r4uA, 3lbgA, 2fubA, 1xxjB, 1r56B, 1ws2D | 2p23A | 3k8kA | 3dkxA, 2cx6A 2p62A, 2za4B 2h9zA 2agh_C |
| 11 | T0579 | 2KY9 | 124 | 3cmeQ, 2ckkA, 3cd6Q, 1vbvA, 3g71Q, 1vqmQ, 3cpwP, 1vq5Q, 2otjQ, 2dppB,**2qqrA** | 1pk6A | **2qqrA** | **2qqrA** |
| 12 | T0586 | 3NEU | 125 | **3by6D,** 2du9A, 2wv0A, **2ek5C**, **3ic7A,** 1h9tB, 3eetA | 1v4rA | **3ic7A** | **3by6A,** **2ek5A** **3ic7A** ,2wv0A |
| 13 | T0598 | 3NJC | 161 | **2osoA**, 2osdA, 1wc8A, 1wc9A, 3cueD, 2j3wE, 2z9eF, 2z9fA, 2cfhC | 1eayA | **2osdA** | **2osoA** |
| 14 | T0602 | 3NKZ | 123 | 1wdzA, 1y2oA, 2br9A, **3a7mA,** 1ciiA 2x0lA, 2xasA, 2iw5A, 2c63C, 2uxxA | 2ohgA, 1b74A | **3a7mA** | **3a7mA** 3h3mA |
| 15 | T0604 | 3NLC | 549 | 1gpeB, 1cf3A, 1galA, 3fimB, 1ju2B, 2igkG, 2igoG, 3k4bA, 3bg6C, 1tzlA, 3bg7B | 1w4xA, 3gwdA | 2i0zA | 1y0pA, 3k30A 2gmhA, 2bs2A |
| 16 | T0605 | 3NMD | 72 | 2fyzE, 2ch7B, 1nknB, 1x7aB, 1jccC, 1ic2D, 1gk4B, 1favA, 1ciiA 1urqB, 2w6aB | 1s721, 1s1iy | 1zxaA | 1ykhA, 1zxaA 1useA |
| 17 | T0606 | 3NOH | 169 | 1olaA, **2nooA,** 1uiuB, 3e3kB, 1b3hA, 1ztyA, 1b4hA, 2olbA, 1b5iA, 1jevA | 1vr5A, 1zuoA | 1l9uD | **2nooA** |
| 18 | T0625 | 3ORU | 233 | **3di4B,** 2vovA, 2qs4A, 2uyaA, 1fxzB, 2uybA, 2vowA, 1l3jA, 1uw8A, 2evxA | 2ofcA, 1gzrB, 1yezA | **3di4A** | **3di4A** |
| 19 | T0628 | 3NUW | 295 | 1x9jB, 1sazA 2iirG, 1x9jD, 2cgkA, 1x3nA, 2e1yA, 1x3mA, 3bp8A, 3khyB | 1hxpA | 2gtdA | 1huxA, 3hz6A |

**(c)**

|  | TARGET | PDB native | Best Templates used in CASP2010 | # of SCOP 1.65 domains | # of PSSMs generated from the SCOP domains |
| --- | --- | --- | --- | --- | --- |
| 1 | T0529 | 3MWT | **2guiA**, 2idoA, 1j54A, 1j53A, **2d5rA** , **1wljA**, 2p1jA**,** 3hfxA, 3kk2A, 3ithA | **3** | **3** |
| 2 | T0544 | 2L3W | 2ffhA, 3cucA, 2hpsA, 1sl8A, 3iytA, 1z6tA, 1df0B, 3glfF, 2x38A, 3iukA | 0 | 0 |
| 3 | T0558 | 3NO2 | 3fvzA, 3li3A, 2gvuA, 3hlhB, 2gvwA, 3fw0A, 2iatA, 3hliC, 3elqA, 3g4hA, **1q7fA** | **1** | **3** |
| 4 | T0562 | 2KZX | 2qq4F, **1su0B**, 3lvlA, 3lwxA**,** 3hrdG, 1n63C, 1n5wC, 1n60C, 1n61C, 1ffvF | **1** | **1** |
| 5 | T0564 | 2LOC | 3hs0C, 1h9mA, 1h9jA, 3gd7A, 1h9rA, 1h9sB, 2r6gB, 1o7lB, 1h9kA, 3fh6A | 0 | 0 |
| 6 | T0568 | 3N6Y | 2jhtA, 1qvyB, 1fsoA, 1ft3B, **1kmtA**, 1cc0E, 2jhuA, 2jhsA, 2jhyA, 1fstB | **1** | **1** |
| 7 | T0569 | 2KYW | 3i57B, 3lyyA, 2pz4A, 3eg9B, **1r6vA**, 3hn5B, 1hzlA, **1kbvC**, 1g1oB, 5ttrF | **2** | **1** |
| 8 | T0574 | 3NRF | 2vztA, **1lmiA**, 2vzuB, 2vzoA, 2xo9A, 3cfuA, 1yewI, 2x09B | **1** | **20** |
| 9 | T0576 | 3NA2 | 2j0pA, 3fm2B, 2j0rA, 2oviD, 2ph0A, 1u9tA, 3gyoA, 3fssA, 3gypA, 3dbaA | 0 | 0 |
| 10 | T0578 | 3NAT | 1xy3F, 2ic0A, 3lwtX, 1r56A, 3f2mA, 1r4uA, 3lbgA, 2fubA, 1xxjB, 1r56B, 1ws2D | 0 | 0 |
| 11 | T0579 | 2KY9 | 3cmeQ, 2ckkA, 3cd6Q, **1vbvA**, 3g71Q, 1vqmQ, 3cpwP, 1vq5Q, 2otjQ, 2dppB | **1** | **2** |
| 12 | T0586 | 3NEU | 3by6D, 2du9A, 2wv0A, 2ek5C, 3ic7A, 1h9tB, 3eetA | 0 | 0 |
| 13 | T0598 | 3NJC | 2osoA, 2osdA, 1wc8A, **1wc9A**, 3cueD, 2j3wE, 2z9eF, 2z9fA, 2cfhC | **1** | **1** |
| 14 | T0602 | 3NKZ | 1wdzA, **1y2oA**, 2br9A, 3a7mA, **1ciiA** 2x0lA, 2xasA, 2iw5A, 2c63C, 2uxxA | **2** | **6** |
| 15 | T0604 | 3NLC | 1gpeB, 1cf3A, 1galA, 3fimB, 1ju2B, 2igkG, 2igoG, 3k4bA, 3bg6C, 1tzlA, 3bg7B | 0 | 0 |
| 16 | T0605 | 3NMD | 2fyzE, 2ch7B, 1nknB, 1x7aB, 1jccC, 1ic2D, 1gk4B, 1favA, **1ciiA** 1urqB, 2w6aB | **1** | **5** |
| 17 | T0606 | 3NOH | 1olaA, 2nooA, 1uiuB, 3e3kB, 1b3hA, 1ztyA, 1b4hA, 2olbA, 1b5iA, 1jevA | 0 | 0 |
| 18 | T0625 | 3ORU | 3di4B, 2vovA, 2qs4A, 2uyaA, 1fxzB, 2uybA, 2vowA, 1l3jA, **1uw8A**, 2evxA | **1** | **1** |
| 19 | T0628 | 3NUW | 1x9jB, **1sazA**, 2iirG, 1x9jD, 2cgkA, 1x3nA, 2e1yA, 1x3mA, 3bp8A, 3khyB | **1** | **2** |

**(d)**

|  | TARGET | PDB native | Residues | Best Templates used in CASP2010 | WAC Labs | FFAS03n | HHpredA |
| --- | --- | --- | --- | --- | --- | --- | --- |
| 1 | T0531 | 2KJX | 65 | 3kstA,1kq6A,2uugC,2uzxB,1shyB,1udiI,1gd5A,3hd8C,1o7kC,3en2A | 1wc9a | 2fug_3 | 1v8hA, 2oxgZ 1p1pA, 1zt3A 1o65A |
| 2 | T0534 | 3N8U | 384 | 1x8zB,1x91A,1cc1L,1sj8A,1h2aL,1h2rL, 1x8zC,1ubuL,1wujL, 3eb7A | 1j8uA, 1p9kA, 2o5hA | 2uv8A | 2o1eA, 3iwpA 2vs0A, 2prsA 3hshA, 3favB |
| 3 | T0537 | 3N6Z | 381 | 2pyhB,2vbmA,2vjiA,2vbeA,2pygB,1oflA,1dboA,1dbgA,1pclA,2qx3A | 1jpmA, 1ttzA | 2dfsA | 2qy1A, 1pe9A 1rmgA, 1xg2A 1gq8A, 1ia5A |
| 4 | T0550 | 3NQK | 339 | 2fwsA,2qvkA,**3eadB,**3e9uA,2dpkA,3ginB,2qvmA,1b9kA, 2uy6A,2vj0A | 1zjcA, 2ayiA | 3ijeA | **3eadA,** 3l4qC 2jnaA, 3e9tA 3fsoA |
| 5 | T0553 | 2KY4 | 141 | 1po5A,1sl8A,2fdwB,1o87A,2pg7B,1lxyA,2j46A,3k3fA,2x49A,1z10B | 1m2xA, 1k03A, 1x86A | 3h0gA | 3k44A, 2a61A 2zfdA |
| 6 | T0571 | 3N91 | 344 | 2fwsA,3e9uA,3fsoA,3h6aA,3h3iC,3fq4A,2qvkA,2qvmA,3ginB,2dpkA | 2bi7A | 3h09A | 1ms9A, 3e9tA 3flpA, 3eadA |
| 7 | T0608 | 3NYY | 279 | 2hsiB, 2b13A, **1qwyA**, 2b0pA, 2gu1A, 2b44B, 3it7A, 3it5A, 3csqD, 2gprA | 2ccvA, 3ia3A, 2hsiA | **1qwyA** | 2gu1A, **1qwyA** 1z65A, 3it5A |
| 8 | T0616 | 3NRT | 103 | 1setB,1sesB,2dq3A,1j46A,1evkA,3c7nA,1sryB,1serA,2ktmA,2pv3B | 1sdoA | 1epwA | 3hv8A, 1wyqA 2vy4A, 2cpgA |
| 9 | T0618 | 3NRH | 182 | 3iq1B,2chpC,2q52A,2cf7G,1umnC, 2fjcJ, 2q4fA, 3hx2r, 3jq1A, 2e0aB | 1xn9A, 3looA,3gd5A | 1i5pA | 2ddhA, 2rklA 2jxfA, 2k42B 3kyjA |
| 10 | T0621 | 3NKG | 172 | 1m4xA, 1m3yC, 3kk5D, 1j5qB, 2cdpC, 2cdoC, 1nqjB, 1ciyA, 2o8oA, 3gbwA | 2r4j1, 1ldlA, 2k8yA | 2bgoA | 1cf1A, 2vzpA 1f86A, 2w87A |
| 11 | T0624 | 3NRL | 81 | 1ha3A, 1ob5A, 1aipA, 2c78A, 2ox7A, 1tttA, 1mj1A, 1fujC, 1exmA, 1xb2A | 2v1cC, 1wh2A | 1e6eB | 2zzeA,1bw3A 3bk5A |
| 12 | T0540 | Cancel | - | - | - | - | - |
| 13 | T0556 | Cancel | - | - | - | - | - |
| 14 | T0561 | Cancel | - | - | - | - | - |
| 15 | T0614 | Cancel | - | - | - | - | - |
| 16 | T0631 | Cancel | - | - | - | - | - |
| 17 | T0633 | Cancel | - | - | - | - | - |
